# Prevalence of Diabetes Mellitus and Its Associated Factors in Bangladesh: Application of Two-level Logistic Regression Model

**DOI:** 10.1038/s41598-020-66084-9

**Published:** 2020-06-24

**Authors:** Ashis Talukder, Md. Zobayer Hossain

**Affiliations:** 1 Statistics Discipline, Khulna University, Khulna, 9208 Bangladesh; 2 Development Studies Discipline, Khulna University, Khulna, 9208 Bangladesh

**Keywords:** Risk factors, Medical research

## Abstract

This study intends to explore the prevalence of diabetes mellitus (DM) and its associated factors in Bangladesh. The necessary information was extracted from Bangladesh Demographic and Health Survey (BDHS) 2011. In bivariate analysis, Chi-square test was performed to assess the association between selected covariates and diabetes status. A two-level logistic regression model with a random intercept at each of the individual and regional level was considered to identify the risk factors of DM. A total of 7,535 individuals were included in this study. From the univariate analysis, the prevalence of DM was found to be 33.3% in 50–54 age group for instance. In bivariate setup, all the selected covariates except sex of the participants were found significant for DM (*p* < 0.05). According to the two-level logistic regression model, the chance of occurring DM increases as age of the participants’ increases. It was observed that female participants were more likely to have DM. The occurrence of DM was 62% higher for higher educated participants, 42% higher for the individuals who came from rich family and 63% higher for the individuals having hypertension. The chance of developing diabetes among overweighed people was almost double. However, the individuals engaged in physical work had less chance to have DM. This study calls for greater attention of government and other concerned entities to come up with appropriate policy interventions to lower the risk of DM.

## Introduction

The epidemic of the century-Diabetes Mellitus (DM), often referred as simply diabetes, is a set of metabolic disorder or syndrome recognized as chronic hyperglycemia (presence of high blood sugar) occurs due to imperfections in insulin action, insulin secretion, or both^1,2^. It is now prevalent across the world with multiple complications^3–5^. In 2014, around 422 million people were affected by DM and 1.5 million deaths were reported in 2012^6,7^. According to WHO report, 2016, 8% (12.88 million) of total population of Bangladesh was affected by diabetes whereas 3% of total deaths of all-ages occurred due to diabetes^8^. An increasing rate in the prevalence of DM among the Bangladeshi populace is also observed over time^9^.

Diabetes may result in a wide range of physiological as well as psychological problems including sexual disorder^10^. The lower sexual functions or dysfunctions termed as loss of libido may be observed in both females and males as a consequence of DM^11–18^. In addition, severe vision loss, acute renal diseases which may require dialysis or kidney transplant, myocardial infarction otherwise known as heart attack, cerebrovascular diseases like stroke, and hypertension are markedly observed^19–27^. Due to the intensity of the adverse effects of diabetes, it is important to find out the determinants to address the issue in order to contribute to improving country health situation.

Diabetes mellitus (DM) is a disease that relies on many factors and may vary over time and region. Accordingly, it requires to be screened on a constant basis. Previously, many studies explored the risk factors of DM by utilizing several statistical models. Among these models, a single level logistic model was very popular. However, the single level model depends on some strict assumptions which may not be possible to follow all the time, specifically, when someone is dealing with a dataset having hierarchical formation. Alternatively, one can apply a two-level regression model. Thus, our study intends to explore the prevalence of DM and its possible risk factors in Bangladesh by applying a two-level regression model since the necessary information was obtained from Bangladesh Demographic and Health Survey (BDHS) 2011 that follows a hierarchical structure.

## Materials and Methods

### Data Collection

This study utilized the BDHS 2011 dataset, which was collected by the National Institute of Population Research and Training (NIPORT) with the collaboration of Mitra and Associates (Bangladesh) and ICF international (USA)^28^. In this survey, a total of 83,731 household members were taken from 17,141 households by applying a two-stage stratified cluster sampling^28^. Although BDHS 2014 data are available, the necessary information for conducting this study such as fasting plasma glucose (FPG) is not available in BDHS 2014 and is very much accessible in BDHS 2011 survey. In 2011 survey, there were 3734 men and 3831 women aged from 35 years and above who participated in FPG measurement^28^.

### Dependent variable

For measuring fasting plasma glucose (FPG), the BDHS 2011 utilizes the cut-off points recommended by WHO^29^. If FPG ranges from 3.9 mmol/l (70 mg/dl) to 6.0 mmol/l (108 mg/dl), it was said to be normal^29^. The FPG value within the range between 6.1 to 6.9 mmol/l (110–124 mg/dl) was considered as pre-diabetic, and the FPG greater than or equal to 7.0 mmol/l (126 mg/dl) was said to be diabetic^29^. In this study, a participant was identified as a diabetic patient if his/her FPG value was greater than 6.1 mmol/l (110 mg/dl)^30^.

### Independent variables

A set of categorical explanatory variables was selected to fit the two level regression model. The selected variables were divided into two parts: level-1 variables and level-2 variables. At the level-1, we considered: sex (male, female), level of education (no education, primary education, secondary education, above secondary education), wealth index (poor, middle, rich), BMI (body mass index) [thin, if BMI is less than 18.5, normal, if BMI ranges from 18.5 to 24.9, overweight, if BMI is greater than 24.9] and working status (desk-work, physical work). The working status was used as a proxy variable for measuring physical activity^31^. Respondents were considered to be engaged in physical activities if his/her work liability includes physical activity related works^31^. This group includes agricultural worker, farmer, fisherman, cattle raising, poultry raising, rickshaw driver, road building, brick breaking, construction worker, domestic servant, boatman, beggar and factory worker^31^. The physically inactive group consists of land owner, unemployed/student, carpenter, tailor, doctor, nurse, dentist, lawyer, accountant, teacher, family welfare visitor, businessman, housewife, religious leader and retired person^31^. The level-2 variable includes region of residence which was classified as urban and rural.

### Statistical analysis

The relationship between chosen covariates and DM was analyzed by performing both bivariate and multivariate investigations. In the bivariate arrangement, chi-square test was utilized to identify the relationship between response and explanatory variables. Weighted prevalence of diabetes among Bangladeshi people was also calculated. In the multivariate arrangement, we applied a two-level logistic regression model to examine the risk factors of DM by reducing the regional effect that exists in the dataset. However, intra-class correlation coefficient (ICC) should be calculated prior to the application of any two-level model^32^. The mathematical form to calculate the ICC is

$$ICC=\frac{var({V}_{0j})}{var({V}_{0j})+({\pi }^{2}/3)}$$

Where, $\,var({V}_{0j})$ represents the variance of random intercept (variance of level-2). The range of ICC varies from 0 to 1. A two-level regression model is applicable, if the ICC is greater than 0^33^.

The term $var({V}_{0j})$ should be calculated from two-level empty model. The functional form of the two-level empty model is-

$$logit({\pi }_{ij})={\alpha }_{0}+{V}_{0j}$$

in which $logit\,({\pi }_{ij})=P({Y}_{ij}=1)$, ${\alpha }_{0}$ represents the intercept of fixed effect and ${V}_{0j}$ represents the level-2 residual^33^. Finally, the mathematical form of the two-level random intercept model is-

$$logit({\pi }_{ij})={\alpha }_{0}+{\beta }_{10}{x}_{ij}+{\beta }_{01}{X}_{j}+{V}_{0j}$$

where ${x}_{ij}\,$and *X*_*j*_ represent the level-1 and level-2, respectively, whereas *β*_10_ and *β*_01_ represent the fixed effects of the level-1 and level-2 variable, respectively^33^.

### Ethical Approval

This study depends on secondary dataset collected by NIPORT (Bangladesh) and MEASURE DHS. Every legitimate strategy was performed including human participants as per the moral norms of the national research committee and with the 1964 Helsinki Declaration and its later amendments or comparable ethical standards^28^. The information records are unreservedly accessible from the website: www.dhsprogram.com. We got approval from the DHS program for utilizing the applicable datasets for this investigation. Ethical clearance for the BDHS 2011 data collection was taken from the ICF International’s IRB (Institutional Review Board)^28^. This survey confirmed international ethical standards of anonymity, confidentiality, and informed consent^28^. A letter of data authorization was taken from the Demographic and Health Surveys (DHS) Program, ICF International. The authority of Statistics Discipline in Khulna University, Bangladesh additionally gave us the chance to direct the examination.

### Informed consent

Informed consent has been taken from all participants associated with this research.

## Results

The background characteristics of study participants by diabetes status and weighted prevalence of diabetes mellitus with 95% confidence interval were displayed in Table 1. It was observed that the prevalence of diabetes increases with increasing age of the participants. The prevalence was almost equal among female (32.4%) and male (32.5%). Considering the education level, the higher rate of DM was observed among higher educated participants. Subsequently, the rich group had highest prevalence of diabetes (36.7%) than their counterparts. Prevalence of diabetes was higher in urban area (35.9%) than the rural area (30.8%). In our study, the prevalence rate of DM was higher among thin (31.6%), overweighed (48.2%), participants having hypertension (23.2%) and not engaged in physical work (32.8%). Except the sex of the participants, all the selected covariates were found to be statistically significant (p < 0.05).

**Table 1:** Background characteristics of study participants by diabetes status and weighted prevalence of diabetes mellitus with 95% confidence interval (CI).

Covariates	Diabetes status		p-value	Prevalence (95% CI)
	Non-diabetes. n (%)	Diabetes. n (%)		
**Age category (year)**				
35–39	1017 (71.2)	412 (28.8)	<0.001	27.2 (26.2, 28.1)
40–44	909 (69.5)	399 (30.5)		29.4 (28.4, 30.4)
45–49	795 (66.5)	400 (33.5)		32.1 (32.1, 34.1)
50–54	667 (65.6)	349 (34.4)		33.3 (31.9, 34.0)
55–59	426 (61.6)	265 (38.4)		37.1 (36.1, 38.2)
60–69	671 (64.3)	372 (35.7)		37.3 (36.2, 38.4)
≥70	561 (63.5)	322 (36.5)		38.1 (37.1, 39.2)
**Gender**				
Male	2503 (67.0)	1231(33.0)	0.558	32.5 (31.4, 33.5)
Female	2543 (66.4)	1288(33.6)		32.4 (31.4, 33.5)
**Education level**				
No education	2384 (69.5)	1047 (30.5)	<0.001	29.4 (28.4, 30.4)
Primary	1377 (66.1)	706 (33.9)		33.7 (32.6, 34.7)
Secondary	915 (65.2)	489 (34.8)		36.1 (35.1, 37.2)
Higher	370 (57.2)	277 (42.8)		40.3 (39.2, 41.3)
**Wealth index**				
Poor	1744 (69.0)	783 (31.0)	<0.001	30.2 (29.2, 31.2)
Middle	1717 (68.3)	796 (31.7)		30.7 (29.7, 31.7)
Rich	1585 (62.8)	940 (37.2)		36.7 (35.6, 37.8)
**Place of residence**				
Urban	1607 (64.6)	882 (35.4)	<0.01	35.9 (34.9, 37.0)
Rural	3439 (67.8)	1637 (32.2)		30.8 (29.7, 31.8)
**BMI**				
Thin	1062 (66.4)	538 (33.6)	<0.001	31.6 (30.6, 32.6)
Normal	2075 (69.4)	915 (30.6)		29.4 (28.4, 30.4)
Overweight	325 (50.1)	324 (49.9)		48.2 (47.1, 49.3)
**Hypertension**				
Yes	1292 (80.0)	322 (20.0)	<0.001	23.2 (22.3, 24.1)
No	3402 (72.3)	1305 (27.7)		19.2 (18.5, 20.2)
**Physical activity**				
Yes	1290 (69.3)	572 (30.7)	<0.01	31.0 (29.9, 32.0)
No	3726 (65.9)	1928 (34.1)		32.8 (31.7, 33.8)

In multivariate analysis, at first, we estimated a two-level empty model to obtain the value of intra-class correlation (ICC) within the divisions. The Table 2 is showing the value of ICC which is 0.019, suggesting that the two-level regression model can be applied to identify the risk factors for diabetes mellitus. The estimates of two-level model were displayed in Table 3. From this table, it was identified that the intercept of random part was statistically significant (p < 0.001), representing that the DM varies among divisions of Bangladesh. From fixed effects, we observed that the chance of having diabetes increases with increasing age of the participant (OR = 1.919, p < 0.01). It was also interesting to find that females were more likely to have diabetes (OR = 1.468, p < 0.001) than males. The occurrence of diabetes was 62% higher for higher educated participants (OR = 1.617, p < 0.001). Compared to the participants from middle income family, the odds of developing diabetes was 42% higher for the individuals who came from the rich family (OR = 1.420, p < 0.01). It was also found that the chance of developing diabetes among overweighed individuals was almost double (OR = 2.121, p < 0.001) than normal weighted people. Moreover, the chance of developing diabetes was higher among persons with hypertension (OR = 1.629, p < 0.001). However, the individuals engaged in physical work had less chance (OR = 0.972, p < 0.05) to develop diabetes compared to their counterpart.

**Table 2:** Parameter estimates of two-level empty model with intra class correlation coefficient (ICC).

Covariates	Estimate	Odds ratio	p-value
**Fixed effect**			
Intercept	−0.682	0.505	<0.001
**Random effect**			
${\delta }_{u0}^{2}\,$(Intercept)	0.064	—	<0.001
**Intra class correlation coefficient (ICC)**			
$${\rm{ICC}}=\frac{0.064}{(0.064+3.289)}=0.019$$

**Table 3:** Odds ratios with fixed and random effects of selected covariates for the diabetes status of Bangladesh obtained from two-level random intercept logistic regression model.

Covariates	Estimate	Odds ratio	p-value
**Fixed effects**			
Intercept	−1.023	0.359	<0.000
**Age category (year) (ref**: **35–39 age group)**			
40–44	−0.190	0.826	0.482
45–49	0.180	1.197	0.511
50–54	0.307	1.359	0.188
55–59	0.563	1.755	<0.05
60–69	0.395	1.484	<0.1
≥70	0.652	1.919	<0.01
**Gender(ref: Male)**			
Female	0.348	1.468	<0.001
**Education level (ref: No education)**			
Primary	0.099	1.104	0.201
Secondary	0.049	1.050	0.607
Higher	0.481	1.617	<0.001
**Wealth index (ref: Middle)**			
Poor	0.101	1.106	0.387
Rich	0.351	1.420	<0.01
**Region of residence (ref: Rural)**			
Urban	0.128	1.136	0.243
**BMI (ref: Normal)**			
Thin	0.109	1.115	0.117
Overweight	0.752	2.121	<0.001
**Hypertension (ref: No)**			
Yes	0.488	1.629	<0.001
**Physical activity (ref: No)**			
Yes	−0.028	0.972	<0.05
**Random effects**			
${\delta }_{u0}^{2}\,$(Intercept)	0.082	—	<0.001

## Discussion

In our study, we observed that the prevalence of DM was more among old age group. Different studies also found that the probability of diabetes was higher with increased age^34–40^. This may occur due to deficiency in insulin secretion following weaker pancreatic function of old people^41^. In addition, the demand of insulin may increase in the human body in certain context or improper utilization of insulin by the body among aged people^42,43^. The suggestive actions for older adults were maintaining blood pressure, blood sugar through improving lifestyle^44–51^.

Relation between gender and prevalence of diabetes mellitus showed a mixed result and geographical location has been found as a key determinant. Though we found almost same prevalence of diabetes in both sex, Gale and Gillespi (2001) found that females with non-European origin and males from European origin were more prone to have diabetes^52^. Hilawe *et al*. (2013) mentioned that the prevalence of DM was relatively higher in men from Middle or Eastern Africa whereas in women from Southern Africa or among respondents from Indian ethnicity^53^. Aregbesola *et al*. (2017) found a higher prevalence of diabetes risk in males^54^. Another study found that the risk of developing diabetes was higher among Caribbean women^55^. A study from China also found a higher prevalence of DM among older women compared with men^56^. In US context, the prevalence of DM was relatively lower among women compared to men between 2013–2016^57^.

Lower educational attainment puts both male and female at a high risk of developing diabetes^58–60^. An inverse association between educational attainment and development of diabetes was observed in different studies^61–63^. A study revealed that low educational level was significantly related to poor glycaemic control for people having Type 2 DM in Bangladesh^64^. Other studies also demonstrated that higher educational level was independently related to better diabetes knowledge and having optimal glycaemic control through attending diabetes education^65–67^. However, Ali *et al*. (2019) found a somewhat reverse causality that hypertension, diabetes and comorbidity were higher among higher educated individuals^68^. Our study also showed similar result.

Prevalence of diabetes was higher amongst higher socio-economic status groups^69–72^. Thus, higher wealth is an independent risk factor for diabetes^73^. The poor socioeconomic status leaves one with a higher risk of remaining undiagnosed for diabetes^74^. Conversely, the richest have higher probability of being diagnosed with diabetes^75^. Our study also showed higher risk of developing DM among rich income group that supports other findings as they usually are less involved in physical work in Bangladesh.

DM is usually a hereditary disease. In addition, there are many causal and confounding factors that contributed to develop DM such as obesity. Obesity may result in diabetes which is well documented in different studies^76–78^. Hypertension is common among patients with diabetes and DM helps facilitating hypertension^79–82^. Diabetes and high blood pressure complement each other due to the fact that they both contain common physiological traits^83^. Diabetic patients experience increased peripheral artery resistance causing elevated systemic blood pressure.

Epidemic of diabetes is associated with decreasing levels of physical activity and an increasing prevalence of obesity. Priority should be given to promote physical activity in this regard^84–86^. Physical activity reduces the risk of diabetes which is evident in different studies^87–89^. Moderate to high level of physical activity is associated with substantially lower morbidity and mortality in people with diabetes^90^. Physical activity can reduce the risk of diabetes complications^91^. It is important for diabetes management as well^92^. Regular physical activity (PA) is recommended for diabetic patients^93^. If needed, counseling on PA by physicians should be considered in this regard^94^.

## Conclusion

Diabetes has become a major public concern across the world due to its pandemic nature. Bangladesh is not an exception. This study attempted to identify the socio-demographic determinants that condition the prevalence of diabetes mellitus among individuals in Bangladesh. It was observed that age, gender, educational attainment, possession of wealth, obesity, hypertension and level of physical activity were some significant predictors of the prevalence of diabetes mellitus among Bangladeshi adults. This study calls for greater attention of government and other concerned entities to come up with appropriate policy interventions to lower the prevalence of diabetes and associated risks.

## Data Availability

The secondary datasets BDHS 2011 are freely available in the following website: http://dhsprogram.com/data/available-datasets.cfm.
